# Neurofeedback as a form of cognitive rehabilitation therapy following stroke: A systematic review

**DOI:** 10.1371/journal.pone.0177290

**Published:** 2017-05-16

**Authors:** Tian Renton, Alana Tibbles, Jane Topolovec-Vranic

**Affiliations:** 1 Rehabilitation Sciences Institute, The University of Toronto 160–500 University Avenue, Toronto, Ontario, Canada; 2 St. Michael’s Hospital, Trauma and Neurosurgery Program, Toronto, Ontario, Canada; 3 Department of Occupation Science and Occupational Therapy, The University of Toronto, 160–500 University Ave, Toronto, Ontario, Canada; University of Nottingham, UNITED KINGDOM

## Abstract

Neurofeedback therapy (NFT) has been used within a number of populations however it has not been applied or thoroughly examined as a form of cognitive rehabilitation within a stroke population. Objectives for this systematic review included: i) identifying how NFT is utilized to treat cognitive deficits following stroke, ii) examining the strength and quality of evidence to support the use of NFT as a form of cognitive rehabilitation therapy (CRT) and iii) providing recommendations for future investigations. Searches were conducted using OVID (Medline, Health Star, Embase + Embase Classic) and PubMed databases. Additional searches were completed using the Cochrane Reviews library database, Google Scholar, the University of Toronto online library catalogue, ClinicalTrials.gov website and select journals. Searches were completed Feb/March 2015 and updated in June/July/Aug 2015. Eight studies were eligible for inclusion in this review. Studies were eligible for inclusion if they: i) were specific to a stroke population, ii) delivered CRT via a NFT protocol, iii) included participants who were affected by a cognitive deficit(s) following stroke (i.e. memory loss, loss of executive function, speech impairment etc.). NFT protocols were highly specific and varied within each study. The majority of studies identified improvements in participant cognitive deficits following the initiation of therapy. Reviewers assessed study quality using the Downs and Black *Checklist for Measuring Study Quality* tool; limited study quality and strength of evidence restricted generalizability of conclusions regarding the use of this therapy to the greater stroke population. Progression in this field requires further inquiry to strengthen methodology quality and study design. Future investigations should aim to standardize NFT protocols in an effort to understand the dose-response relationship between NFT and improvements in functional outcome. Future investigations should also place a large emphasis on long-term participant follow-up.

## Introduction

In 2011, stroke was identified as the third leading cause of death among Canadians (5.5%, 13 283 deaths), and considered to be the leading cause of neurological disability in Canadian adults [1, 2]. Although stroke occurrence is most common in individuals aged 70 and older, stroke incidence for individuals over the age of 50 has increased by 24% and 13% in individuals over the age of 60, in the last decade [3]. Following a stroke, patients typically enter rehabilitation programs (i.e. physical therapy, occupational therapy, etc.) to address a multitude of physical, emotional and cognitive deficits [4, 5]. Many rehabilitation interventions initiated following stroke primarily target functional motor impairments. In reviewing the literature, few investigations have been published that aim to target cognitive deficits, despite 40% of stroke survivors experiencing a decline in cognitive function (especially memory) following stroke [6].

The brain is a highly complex and organized organ therefore the extent of impairment and deficits that follow stroke are largely dependent on lesion severity and location [7]. Physiologically these impairments are a result of the loss of neuronal circuits and connections linked to the relevant sensory, motor, and cognitive functions [8, 9]. Furthermore, it is thought that the neurological recovery that occurs following a stroke is a direct result of brain plasticity and it’s ability to repair and reorganize [10]. Some evidence exists for the initiation of reparative functions in the brain in as little as a few hours following a stroke [11, 12]. In respect to recovery trajectories following stroke, ninety-five percent of stroke patients reach their peak language recovery within 6 weeks of a stroke, and within 3 months for hemispatial neglect [13, 14]. Deficits that do not spontaneously resolve contribute to the large number of individuals requiring long term care following stroke (i.e. rehabilitative therapy) [15, 16]. Occupational and physical rehabilitation programs target functional and mobility issues however, in addition to these impairments patients experience a wide range of cognitive and neurological deficits. Individuals with impairments of this nature often require cognitive rehabilitation therapy (CRT).

CRT encompasses any intervention targeting the restoration, remediation and adaptation of cognitive functions including: attention, concentration, memory, comprehension, communication, reasoning, problem solving, planning, initiation, judgement, self-monitoring and awareness [17]. CRT can be offered in a variety of settings such as rehabilitation hospitals, community care facilities, private residences as well as the workplace [18]. Although cognitive therapy has been around since the early 19^th^ century, the 1970’s marked the most recent biofeedback movement in CRT [18]. Traditionally used to treat muscular impairments (via electromyography (EMG) feedback) biofeedback has taken on a new form known as neurofeedback therapy (NFT). NFT targets the brain and cognitive functions through the use of electroencephalography (EEG), hence neurofeedback is sometimes referred to as EEG biofeedback [19]. In classical NFT, EEG and brainwave activity is provided as a visual or auditory cue to the user [6]. Using these cues the user can consciously adapt their brainwave activity to reach targeted training thresholds. NFT relies on operant conditioning to stimulate the neuroplastic abilities of the brain [20, 21]. Physiologically stimulating specific band frequencies over damaged areas stimulates cortical metabolism [19]. NFT is also used to counter excessive slow wave activity (i.e. theta waves and sometimes alpha waves) that typically follow stroke [21]. An alternative form of NFT known as *nonlinear dynamical neurofeedback* has also been used to restore homeostasis to the brain. This form of NFT requires no conscious effort from the participant to adapt their brainwaves in any particular direction (i.e. the participant maintains a passive role). Modalities like NeurOptimal® utilize *Functional Targeting* to provide the brain with “… information about itself which allows the brain to assemble it’s own, best organizing strategies moment by moment” [22]. In the context of this review, the studies included herein concern the use of classical NFT only.

To date, NFT has been used extensively to treat cognitive deficits associated with other neurological disorders and illnesses including: mild traumatic brain injury [23], ADD/ADHD [24], Epilepsy [25], Autism Spectrum Disorders [26, 27], Dyslexia [28], Fibromyalgia [29], Depression [30], and opiate additions [31]. Despite promising NFT outcomes within these populations, NFT has not been thoroughly evaluated for use in a stroke population. The aim of this systematic review was to thoroughly evaluate the available evidence pertinent to understanding the effectiveness of NFT as a form of CRT following stroke. To achieve this objective a number of research questions were established to guide this review:

1. Among a stroke population, *how* is NFT utilized to treat cognitive deficits?
2. Among identified NFT interventions targeting a stroke population, what is the *quality* and *strength* of evidence to support the use of NFT as a form of CRT following stroke?
3. Based on the available NFT evidence for use in stroke populations, what *recommendations* can be made for *future research*?

The primary outcome of interest in this review was to identify if cognitive symptom complaints could be ameliorated following the initiation of NFT in a sub-acute and chronic post-stroke population. Secondary outcomes aimed to assess study quality, methodology and strength of support for use of NFT in this population.

### Literature search

An electronic search was conducted using OVID (included Medline, Health Star, Embase/Embase Classic) and PubMed databases. Two separate searches using different search terms were run in each database. Table A in S1 File & Table B in S1 File contain the primary and secondary OVID and PubMed searches. Tertiary searches were conducted using alternative scholarly databases (Google Scholar, The University of Toronto (U of T) Libraries catalogue, The Cochrane Reviews online database and ClinicalTrials.org database) (see Table C in S1 File). Google Scholar was searched twice, first with a broad search (i.e. non-specified cognitive deficits) and then once more using more specific cognitive deficit key terms (similar to the primary and secondary search strategy). The U of T Libraries catalogue was also searched using the specified terms. The Cochrane Reviews online database and ClinicalTrials.org database were also searched as indicated. Finally, 11 of the most relevant scholarly peer-reviewed journals were hand searched. Journals and dates searched are included in Table D in S1 File. Journals were selected using the U of T Libraries catalogue e-journal finder function. Key words (i.e. stroke, neurofeedback, biofeedback, cognitive therapy, etc.) were typed into the search bar to locate journals by title. Additional journals were identified using the citations from the included studies (from the primary and tertiary search strategies). All journals were searched from 2005 onwards until the most current issue to-date available. Tertiary searches and journals were not exported to EndNote (citation management software). Therefore duplicates were not accounted for within either of these searches.

All searches were completed twice; one reviewer completed the search in February/March 2015 (TR) and a separate reviewer completed the same search again in June/July/August 2015 (AT). After completing the searches, the two authors independently reviewed study titles, abstracts and full texts for inclusion. Authors discussed discrepancies until consensus was reached. Both searches included stroke and neurofeedback key terms (and related exploded terms) but used the broad key term “cognitive deficit” (primary search strategy) or specific cognitive deficit key terms (secondary search strategy). Specific cognitive deficit key terms included: attention, memory, perception, neglect, and executive function. Results from both primary and secondary searches (Table A in S1 File & Table B in S1 File only) were exported to EndNote. Duplicates were removed and accounted for as indicated but only within each search, not across the searches (i.e. duplicates were not tracked between primary and secondary searches).

### Study selection

Publications included within this review met the following inclusion criteria:

1. included a stroke population sample
2. delivered CRT via NFT (i.e. biofeedback utilizing brainwaves, EEG)
3. included participants with cognitive deficits (i.e. memory, executive function, speech production or communication, planning, concentration, perception, processing, or attention)

Publications were excluded if they:

1. included a traumatic brain injury population sample in addition to a stroke sample
2. focused on movement, postural, or balance-related deficits
3. used NFT to monitor brainwave activity and not as a treatment
4. did not include a NFT intervention (i.e. editorials or reviews)
5. were not available in English
6. included stimulation (i.e. transcranial magnetic stimulation [TMS]) or a pharmaceutical trial in addition to a NFT protocol
7. was an abstract only or the full article could not be retrieved
8. targeted cognitive deficits as a secondary outcome

Note: Given the limited number of studies that were identified following the initial searches, authors sought to include as many studies as possible in this review. Therefore studies were not considered for exclusion based on study design alone.

### Data extraction

**Fig 1:**
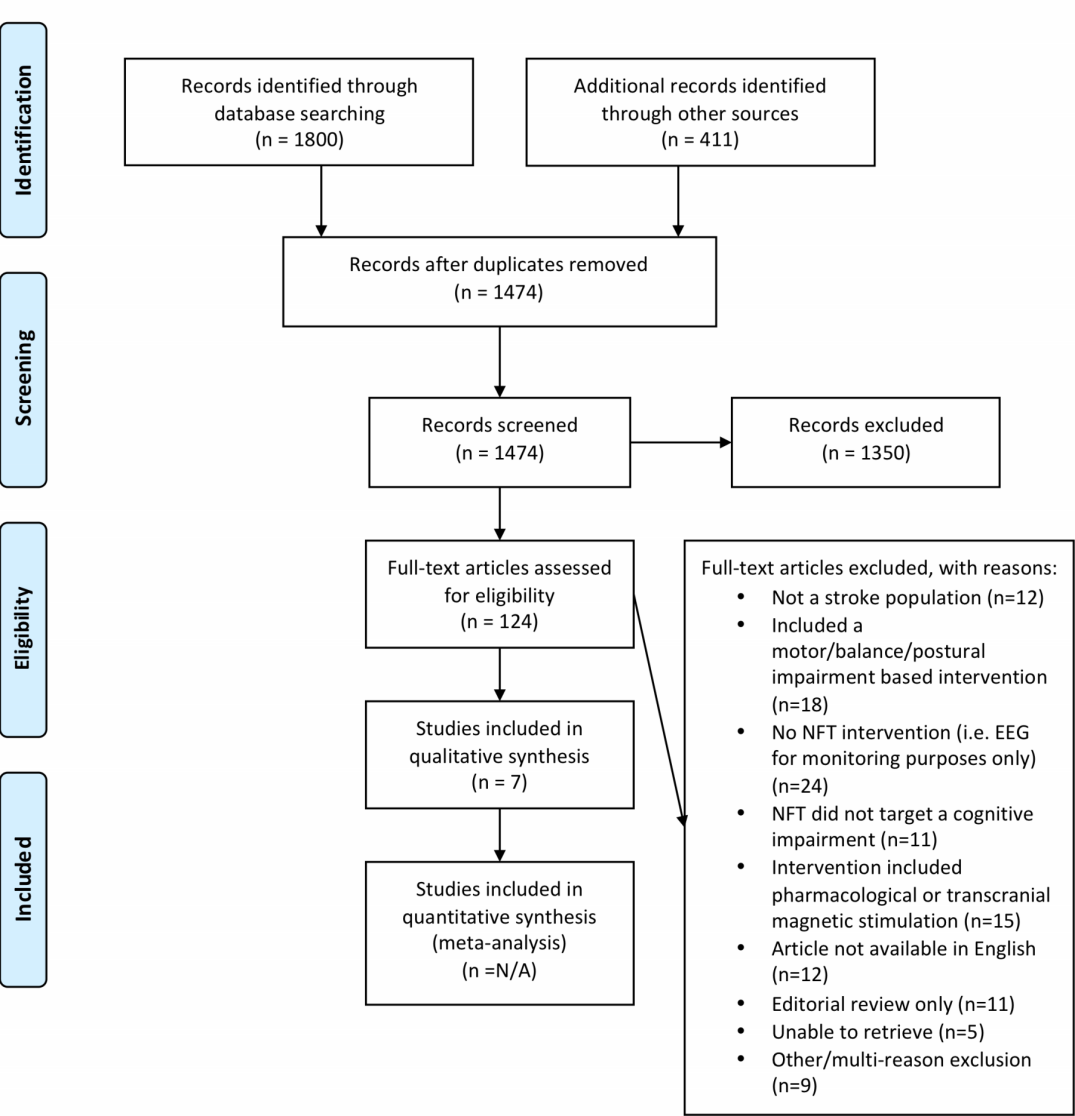
PRISMA flow diagram. Articles identified for inclusion in this review using databases, grey literature and journals. Figure taken from: Moher D, Liberati A, Tetzlaff J, Altman DG, The PRISMA Group (2009). *P*referred *R*eporting *I*tems for *S*ystematic Reviews and *M*eta-*A*nalyses: The PRISMA Statement. PLoS Med 6(7): e1000097. doi:10.1371/journal.pmed1000097.

This review was completed according to the PRISMA statement [32]. Please see S2 File for a completed copy of the PRISMA checklist. Fig 1 contains the PRISMA flow diagram (inclusive of primary, secondary and tertiary search results). A number of systematic review guides were referenced to establish data extraction elements [32–36]. Data extraction was performed systematically by the same two authors who conducted the searches. Articles were read and re-read to ensure points of interest were not missed. Data extraction elements included: study design, participant details (time since stroke, stroke location, cognitive deficits, additional therapy and medications used), outcome measure administration timeline and data collected (i.e. QEEG analysis), NFT training protocol, and key findings. Limitations and bias are further discussed in the discussion section of this review and were not included in data extraction tables. Table 1 includes participant and study design details. Table 2 includes the outcome measures utilized as well as each respective study’s key findings.

**Table 1:** Study design & participants.

Citation	Study Design	Participant Details	Session Details
**Rozelle**	Case Study	Male, 55 yrs	69 Sessions
**1995**		1 yr post CVA	
		SL: left posterior temporal/parietal infarction	EEG: sessions 1–21, 4-5x/week for 15–25 minutes
		AT	
		M	NFT: 22 weeks/48 sessions
		CD: garbled speech, short-term memory loss, tinnitus, fatigue	Sessions 1–6, daily
			4 week break between session 6 & 7
			Monopolar and Bipolar
			Focused Technology F-1000 Biofeedback system
**Bearden**	Case Study	Male, 52 yrs	14 weeks / 42 sessions/ 3x/week then reduced to 2x/week
**2003**		1 yr post CVA	Time per session varied (9 to 14 minutes)
		SL: left hemispheric ischemic infarction	
		AT	Monopolar
		M	
		CD: severely impaired memory and ability to read, upper field visual deficit, inability to learn new material	Thought Technology
			Procomp+/ Biograph biofeedback system
**Doppelmayr**	Controlled Trial	*Experiment 1*:	Experiment 1: 10 days/ 10 sessions, 12 minutes each
**2007**		NFT n = 15	
		Control n = 17	Experiment 2: 10 days/ 9–15 sessions, 16 minutes each
		Mean age: 56 yrs (SD = 13)	
		*Experiment 2*:	Monopolar
		Alpha NFT n = 7	
		RSA n = 6	Procomp+system
		AT (both)	
**Cannon**	Case Study	Female, 43 yrs	26 weeks/52 sessions, 2x/week for 30 minutes
**2010**		1 year post CVA	
		SL: right hemisphere artery embolus	Monopolar and Bipolar
		AT	
		M	NeuroCybernetics
		CD: energy and mood issues, inability to focus, distracted	
**Toppi**	Case Studies	*Participant 1*: Female, 70 yrs	10 sessions @ 25 minutes each, 3 min baseline, six 3 minute training sessions
**2014**		*Stroke Location*: right hemispheric stroke	
			Monopolar
		*Participant 2*: Male, 20 yrs	
		*Stroke Location*: left hemispheric stroke	Modality: not specified
**Mroczkowska**	Case Study	Female, 53 yrs	10 weeks/30 sessions, 3x/week, 40 minutes each
**2014**		~1 month post CVA	
		SL: hemorrhagic stroke in the left hemisphere	Monopolar
		AT	
		CD: anxiety, motivation and cognitive/executive function issues, Broca’s aphasia; speech impairments	Exememory Application
**Cho**	RCT	*NFT* n = 13	6 weeks/30 sessions, 5x/week, 30 minutes each
**2015**		Age– 62.9 yrs (+/-7.2)	
		Lesion (right/left)– 9/4	Monopolar
		*Control group* n = 14	NeuroComp System
		Age– 63.6 yrs (+/-9.3)	
		Lesion (right/left)– 8/5	
		AT	
		CD: visual perception and reductions in cognitive function	

Abbreviations: additional therapy (AT); cerebral vascular accident (CVA); electroencephalography (EEG); Medication (M); Mini Mental State Exam (MMSE); neurofeedback (NF); randomized controlled trial (RCT); respiratory sinus arrhythmia biofeedback training (RSA); Sensorimotor Rhythm (SMR), stroke location (SL).

**Table 2:** Measures used & key findings.

Citation	Measures Used	Key Findings Post-NFT
**Rozelle**	BASRS, BNT, BDAE, AB,	+ speech abilities
**1995**	SCWT, BSI, CCPT, RPM	+ neuropsychological batteries
		+ short-term memory
	Additional: VI, HWS, STR, NA	+ improvement of anxiety and depression
		+ visual tracking and ability to focus
		+ tinnitus cessation
		*tests of statistical significant not performed/not indicated for pre-post measures*
**Bearden**	BSI	+ NA subscales (TMT, WMS, DS). Significant improvements on some
**2003**		+ BSI score
	Additional: RST, NA	+ RST
		*tests of statistical significance were run indicating significant improvements on measures, but p-values were not indicated*
**Doppelmayr**	RBMT	*Experiment 1*: significant effect for time (pre to post) and group (NFT) on RBMT
**2007**		- significant interaction between group and time on RBMT
		= RBMT remained stable over time for the control Relaxation control group
		*Experiment 2*: all groups showed improvements in RBMT over time
		Group and interaction did not reach significance
		*for both studies: tests of statistical significance were run indicating significant improvements on measures, but p-values were not indicated*
**Cannon**	SDC	Symptoms improved from “very problematic” at baseline to “somewhat better”
**2010**		Positive comments from participant: “I’m more with it”, “… more confident”, “people can’t tell I’ve stroked”
		* cessation of anti-depressants @ session 15 *
		*tests of statistical significant not performed/not indicated for pre-post measures*
**Toppi**	RAVLT & CBTT	*Participant 1*:
**2014**		+SMR amplitude
		+ RAVLT & CBTT (p<0.05)
		+ SMT (p<0.05)
		*Participant 2*:
		= SMR amplitude
		= RAVLT & CBTT
		- STM
**Mroczkowska**	BADLI, GKS, BVRT, CTT, MMSE, BDI, RPM, EPQ, TOPS	+ concentration
**2014**		+ visual perception
		+ mood
		+ speech
		*tests of statistical significant not performed/not indicated for pre-post measures*
**Cho**	MVPT	MVPT:
**2015**		+ *NFT*: raw score (p<0.01), time (p<0.001)all values (sig)
		+ *Control*: raw score (p<0.01), FC, time (p<0.01)sig)
		Significant difference between groups (time and score, p<0.05)

Abbreviations: Apraxia Battery (AB); Barthel Activities of Daily Living Index (BADLI); Boston Aphasia Severity Rating Scale (BASRS); Boston Diagnostic Aphasia Examination (BDEA); Beck Depression Inventory (BDI); Boston Naming Test (BNT); Brief Symptom Inventory (BSI); Benton Visual Retention Test (BVRT); Corsi Block Tapping Test (CBTT); Conners Continuous Performance Test (CCPT); cognitive deficit (CD); Colour Trails Test (CTT); Digit Span (DS); Eysenck Personality Questionnaire (EPQ); Goodglass and Kaplan Scale (GKS); Hand Writing Sample (HWS); Mini Mental State Exam (MMSE); Motor-Free Visual Perception Test (MVPT); Neuropsychological Assessment (NA); Rey Auditory Verbal Learning Test (RAVLT); Rivermead Behavioural Memory Test (RBMT); Ravens Progressive Matrices (RPM); Reading Speed Test (RST); Self-Developed Checklist/Short Answer Interview (SDC); Speech Therapy Report (STR); Stroop Colour and Word Test (SCWT); Sternberg Memory Test (SMT); Trails Making Test (TMT); Tools for Optimal Performance (TOPS); Video Interview (VI); Wechsler Memory Scale (WMS). Symbols: + improvement in performance, —decrements in performance, = no change.

### Assessment of methodological quality

Interventions were assessed for level of evidence and methodology quality and strength. Level of evidence was assessed using the Oxford Centre for Evidence-Based Medicine–Levels of Evidence (March 2009) table [37]. Quality assessment was conducted using the Downs & Black Critical Appraisal Checklist [38]. This checklist was pilot tested and then re-assessed, demonstrating improved reliability and validity (criterion validity = 0.90) [38, 39]. Analyses revealed high internal validity (Cronbach alpha > 0.69) on all subscales except external validity which received a Cronbach alpha = 0.54 [38, 39]. Test-retest varied between 0.69–0.90 and inter-rater reliability scores were high (r>0.70) [38, 39]. In a systematic review of critical appraisal checklists, the Downs & Black checklist was ranked as one of the top two scales (out of 182) [40]. The scale contains 27 items and has a maximum score of 32. The scale is broken down into 5 sections: Study Quality (10 items), External Validity (3 items), Study Bias (7 items), Confounding and Selection Bias (6 items), and Study Power (1 item). Item inclusion is scored using “yes” (1 point), “no” (0 points), and “unable to determine” (0 points) responses. Items 5 & 27 offer a “partial” response (1 point, yes = 2 points) and sample size dependent response (score dependent on smallest intervention group size, 0–5 points), respectively.

Each publication was assessed for level of evidence, methodology quality and strength. This was assessed by two independent reviewers (TR and AT) (see Table 3). Level of evidence was assessed using the Oxford Centre for Evidence-Based Medicine–Levels of Evidence table [37]. As mentioned, quality assessment was conducted using the Downs & Black Critical Appraisal Checklist [38]. Following independent evaluation, discrepancies were discussed among reviewers until agreement was met.

**Table 3:** Downs & Black Critical Appraisal Score & Oxford Level of Evidence.

Citation	Report/ Study Quality	External Validity	Study Bias	Confounding Selection Bias	Power	Total Score	Oxford Level of Evidence
	(n of 11)	(n of 3)	(n of 7)	(n of 6)	(n of 5)	(n of 32)	
**Rozelle**	8	0	6	1	0	15	4
**1995**							
**Bearden**	9	0	5	1	0	15	4
**2003**							
**Doppelmayr**	4	1	5	1	0	11	2B
**2007**							
**Toppi**	9	0	4	1	0	14	4
**2010**							
**Cannon**	7	0	5	1	0	13	4
**2014**							
**Mroczkowska**	6	1	4	1	0	12	4
**2014**							
**Cho**	8	1	4	3	5	21	1B
**2015**							

## Results

A total of seven publications with eight studies were eligible for inclusion within this review (see Table 1). One article included two intervention protocols; study designs were identical, except the second intervention incorporated the use of an additional NFT group [6]. Overall, study designs and NFT therapy and training protocols were diverse and heterogeneous in nature. Five of the interventions were case studies [41–45]. Three controlled trials were also included [6, 46]; two were non-randomized and non-blinded (experiment 1 n = 32, experiment 2 n = 17) [6], and one was randomized but non-blinded (n = 27) [46].

### Participants

Three studies included participants in the chronic phase of stroke recovery (i.e. 6 months or more post-stroke)[41–43]. Two studies indicated participants were recruited from an in-patient rehabilitation hospital, possibly implying these participants were still in the sub-acute phase (i.e. 15 days to 6 months post-stroke) [6, 46]. One study included participants whom bordered the sub-acute/chronic phase [46], another included a sub-acute participant [44]. One study did not indicate time since stroke [44]. Participants ranged from 20 to 70 years old [44]. Individuals suffered from various lesions (see Table 1) and exhibited a number of cognitive deficits. NFT interventions directly targeted memory [6, 41, 44], emotional issues [41, 43, 45], concentration and distraction issues [41, 43, 45, 46], fatigue and energy deficits [41, 43], communication and speech deficits [41, 42, 45], visual perception deficits [45, 46] and motivation [45]. Two patients complained of visual tracking deficits, [41, 42] in addition to one of them suffering from tinnitus [41]. See Table 1.

A few of the participants opted to try NFT after already having participated in conventional forms of stroke rehabilitation therapy; additional therapies included speech therapy [41], physical therapy [41, 46], group and individual therapy [42], talk therapy [43] and neuropsychological therapy [45]. Participants in one study completed additional computer training (targeting attention, language, and memory) for 30 minutes immediately prior to their group-specific intervention [6]. Some authors also described the use of medications in addition to NFT use (i.e. for high blood pressure [41, 42] and depression [42, 43]). Authors of one investigation reported the cessation of anti-depressants during the course of the NFT intervention [43].

### NFT protocol

Prior to beginning training, all participants completed a qualitative EEG (QEEG) baseline assessment to identify bandwidth activity. Overall, NFT protocol design and bandwidth training objectives varied between each study. As such, the described neurotherapeutic protocol (i.e. bandwidth thresholds, rewards and inhibited band frequencies) were highly specific within each study (refer directly to each publication for more information). Despite heterogeneity of task objectives and variance of NFT EEG thresholds within each study, the following is a representative example of what a NFT protocol may look like (taken from [42]):

Investigators utilized a 2-fold NFT strategy. The primary objective attempted to reduce theta over P3 (area of maximal dysfunction in the participant). The secondary objective attempted to improve thalamocortical integration over the left central sensorimotor strip (T3 –C3). Visual feedback via computer screen was provided to the participant in the form of a green square (represented theta band activity). In turn, the participant was instructed to keep the green square inside a target box (representing a preset 10-μV threshold). A timer and auditory cue was provided to the participant if they were able to keep theta amplitude within the target box for 250 ms or longer. An additional bar graph display presented electromyography (EMG) activity to the participant. When EMG activity surpassed a preset threshold, the bar graph turned red and inhibited the other visual and auditory feedback. Thresholds were adjusted based on prior participant performance.

Other examples of visual or auditory feedback included a green ellipse presented as a visual representation of brain wave activity that appeared and disappeared and varied in colour intensity depending on preset thresholds [41]. Another study utilized fluctuating bar graphs to represent brain wave activity, rewarding the participant with points if they were able to maintain a certain threshold for 0.25 seconds [6]. For further detail, readers are encouraged to review each study directly.

EEG modality and software utilized to provide bandwidth feedback were specific to each study (see Table 1). Duration (for feedback and baseline data collection) and number of sessions also varied greatly across studies (see Table 1). Number of sessions completed by participants ranged from 8.5 [6] up to a total of 69 sessions [41]. Training ranged from a minimum of 12 minutes [6] to a maximum of 43 minutes per session [42]. Training protocols occurred in as few as two NFT sessions per week [43] up to 10 consecutive days of training [6]. Some NFT protocols included multiple feedback locations ranging from 5 training feedback sites [43] to as few as one feedback site [6, 44]. All paradigms used monopolar feedback (singular site feedback) with the exception of two studies, which included a bipolar feedback component (dual, concurrent site feedback)[42, 43]. See Table 1.

### Key findings & outcomes

The majority of individuals included in the assessed studies demonstrated improvements in their respective pre-post NFT outcome measures. Following NFT, participants noted improvements to memory [6, 41, 42, 44], mood [41–43, 45], concentration [41–43, 45, 46], energy [43], reading and speech abilities [42, 45] and motivation [45]. Despite these positive findings, it is imperative to note that the majority of these studies (with the exception of two studies [6, 46]) lacked a comparator or control group with which to compare NFT treatment outcomes. Although given the chronic stage of recovery that the majority of participants were in upon initiation of NFT, it is unlikely that positive results identified are a product of time alone. Readers should also be aware that due to the heterogeneous nature of the NFT protocol designs, pre-post NFT bandwidth comparison across studies was not completed. Post-NFT outcome measure findings are presented for each study (see Table 2). Note that analysis of EEG and QEEG data was outside the scope of this review and will not be further discussed herein; p-values from each study’s respective wavelength changes pre- to post-NFT initiation are also not reported here. Please refer to the individual publications referenced for more information.

### Quality appraisal findings

The Downs & Black Critical Appraisal Checklist [38] was used to critique the included studies. Total score and scores for each subscale (Reporting, External Validity, Internal Validity- Bias, Internal Validity–Confounding [Selection Bias] and Power) are reported in Table 3. Total scores ranged from 12 [45] to 21 [46] out of a possible total of 32 points. Overall, publications scored poorly within both external and internal validity (confounding–selection bias) categories. Power was also insufficient for the majority of publications (case studies). Oxford Level of Evidence [37] is also indicated for each publication.

## Discussion

A review of the available literature suggests there has been limited investigation into NFT as a form of CRT within a stroke population. Findings from this review provide little support for NFT use as an evidence-based treatment; this is in part due to the limited quality and strength of the interventions conducted. Despite these limitations, modest cognitive improvements were reported among study participants. It is important to note that NFT offers a number of benefits to users including a highly individualized approach to treatment. Integration of pre-post QEEG data collection as part of the NFT protocol allows for an in-depth brainwave analyses specific to the user.

Timing of NFT treatment initiation in the included studies is also noteworthy. Previous literature has indicated that functional recovery typically occurs in ~95% of stroke patients within the first 12.5 weeks (~3 months) of a stroke event [47]. Despite surpassing this recovery window, participants included in the studies captured in this review exhibited cognitive improvements [41–43]. This would indicate that improvements are not likely a result of time alone (i.e. it is unlikely that symptoms would resolve spontaneously beyond the recovery window that is typically observed in a stroke population). To enhance interpretation of the effect of NFT on improving cognitive deficits following stroke, it is important to consider the phase of recovery of the sample captured herein. Referring to the greater stroke literature, there are some discrepancies surrounding how acute, sub-acute and chronic phases of recovery are defined. One source identified the sub-acute phase ranging from 1 week to 3 months post-stroke, defining chronic as greater than 3 months [48]. Other sources defined the acute phase from time of stroke until 14 days, sub-acute from 15 days to 6 months, and chronic as greater than 6 months [49–51]. Based on this consensus, sub-acute and chronic recovery were defined using this precedence. Although unclear or undefined within two investigations [6, 44], and defined in two studies [45, 46] (3 months to 1 year post-stroke and ~ 1 month post-stroke respectively), four investigations may have included participants in the sub-acute recovery phase. Among these investigations, improvements were identified within each study’s respective neuropsychological assessments used pre- post-NFT initiation. When considering the results from these four particular studies, it is more likely that the effect of time would have contributed to the identified improvements, rather than the effect of the NFT alone; this further decreases the strength of evidence in support of NFT as a form of CRT following stroke. To this extent and to enhance interpretation of results, future investigations should aim to clearly define the stage of recovery that their sample falls within (i.e. use of standardized and objectively defined recovery phases across studies will aid with comparisons of groups). Furthermore, future studies should aim to compare the effect of NFT interventions between sub-acute and chronic stroke populations to better understand the interaction between time since stroke and efficacy of NFT. However, the heterogeneous nature of stroke further complicates sub-acute and chronic stroke group comparisons and should be considered when interpreting results (i.e. irrespective of time since stroke, type of stroke, lesion location and severity will likely impact efficacy of NFT).

It is also important to note that many of the participants had received prior therapy (i.e. speech therapy [41, 45], group and individual therapy [42, 45], and talk therapy [43]) and failed to see any improvement in their deficits. NFT offered participants a novel therapeutic intervention that to some extent they were highly motivated to pursue. Although it is likely that NFT may be what led to the observed cognitive improvements, it is unlikely that the NFT intervention alone is responsible for these results. Specifically, contextual motivation (i.e. motivation to complete a given task is dependent on the context) or situational motivation (i.e. motivation to complete a given task is dependent on the point in time an individual is being asked to complete it) within each participant may have played a large role in improving their respective cognitive deficits. In regards to contextual motivation, NFT was a novel therapy (i.e. predominant use of a computer-based program) for which participants may have been more likely to actively engage in, as compared to engagement in more traditional forms of therapy (i.e. physiotherapy or occupational therapy). In respect to situational motivation, being that the majority of individuals included in these studies were considered to be in the chronic recovery stage, and in addition to their prior participation in other forms of therapy, it is likely that these individuals were willing to try anything to improve their persistent cognitive complaints. Furthermore, any initial gains identified immediately following initiation of NFT would also serve to positively reinforce their engagement in NFT. In summary, a positive feedback loop would have also contributed to a participant’s sustained engagement in the NFT program.

Any conclusions regarding the dose-response of NFT (i.e. greater number NFT sessions leads to better results) is cautioned. Investigations provided a low level of evidence and satisfactory methodological design. Despite the 20 year span between the oldest [41] and most recent study [46], methodological quality and level of evidence had not substantially improved. Research on NFT as an intervention for post-stroke cognitive impairments may be limited by: excessive technology costs, time-consuming and complex intervention set-up, strict training location specifications or cumbersome personnel training requirements. Limitations of NFT training in this respect were not described in any of the studies reviewed. Furthermore, many (if not all) of the publications failed to describe the longevity or maintenance of any cognitive improvements associated with NFT therapy; long-term follow-up was not included in the majority of study protocols, with the exception of one study which conducted a qualitative interview 1 month following the completion of the NFT intervention [43]. Bias or confounding were inherent in most studies as participants had either tried multiple forms of therapy prior to NFT initiation, were non-blinded to the potential effects of NFT, or were not randomized equally into their respective experimental groups. Confounding factors (i.e. use of prior therapy or medication use) were mentioned in a few studies however authors did not discuss the implications of these factors on treatment outcomes [41–43, 45].

A number of limitations are present within this review. Although search execution and data extraction was completed independently by two reviewers (TR, AT), it is possible that studies meeting inclusion criteria were missed. Furthermore, some time has since past from the date of the last search (August 2015); it is possible that new studies have been completed and published, and have not been included in this review. Subsequent systematic reviews should focus on identifying publications from August 2015 onwards. NFT was also unknown to the reviewers prior to beginning this systematic review, and reviewers lacked clinical experience and expertise in administering and interpreting NFT (Q)EEG data. On this basis, it would be inappropriate for these particular reviewers to conduct a quantitative meta-analysis of the data presented here. Furthermore, use of the Downs & Black Critical Appraisal Checklist was found to be both difficult and time consuming. Some literature indicated that it’s use requires considerable epidemiological expertise [52]. Although the measure was novel to both reviewers, all outstanding questions were directed to the authors of the scale for further clarification. Lastly, NFT protocols were highly specific to each study (i.e. feedback location, number of sessions, training task involved, etc.) and targeted diverse cognitive domains. Therefore cross-study comparison with the objective of formulating a general conclusion about overall NFT impact was not feasible due to the large variation among NFT protocols. It should also be noted that the studies included herein utilized a variety of neuropsychological assessments to gauge and monitor cognitive improvements following the initiation of NFT; although each investigation collected their respective measures pre- and post-intervention, few performed or reported p-values (with the exception of two studies [44, 46]). This further convolutes interpretation of the pre-post NFT changes identified within each study, thus making it difficult for the authors of this review to make any conclusive statements about the effectiveness of NFT in a stroke population. Overall, heterogeneous NFT protocols and participants greatly limit the generalizability of these findings to a greater stroke population.

### Caution for neurofeedback

Adverse negative effects for NFT were not detailed within any of the publications captured in this review and are sparsely recorded within the literature. Only one study described decrements in participant performance following NFT [44]. Iatrogenic (relating to illness caused by medical examination or treatment) and over training effects are thought to occur when NFT is administered inappropriately [21]. Negative effects include: emotional lability, vocal tics, deterioration or loss of improvement, regression, muscle twitches, somatic symptoms, incontinence, enuresis, mental fogginess and cognitive inefficiency, sleep disturbance, fatigue, seizure, anxiety/rage/depression and slurred speech [53]. Ethical considerations have limited the number of studies investigating or attempting to provoke iatrogenic effects from NFT treatment [53]. Additionally, some individuals as compared to others may be highly sensitive to over-training. Anecdotal evidence has indicated some participants could only stand 2 to 3 minute bouts of NFT within an hour long session [54]. One expert suggests constant monitoring for task-fatigue in all participants during NFT administration [54]. Recommendations also include an individualized approach (i.e. pre-NFT EEG analysis) to subsequent NFT protocol development [21].

Recent attention on NFT has contributed to an influx in unlicensed and untrained operation of biofeedback equipment [53, 55]. For this reason individuals are encouraged to seek Biofeedback Certification International Alliance (BCIA) (formally Biofeedback Certification Institute of America) accredited professionals. More information can be found on the BCIA website: www.bcia.org. Furthermore, a Standards of Practice Committee of the International Society of Neurofeedback and Research (ISNR) has developed a number of guidelines to ensure continued patient safety throughout NFT treatment [55, 56]. Guidelines and standards of practice include industry regulations such that NFT administrators be: i) licensed as per regulations in their respective countries/states/provinces, ii) keep up-to-date on current developments in the field, iii) keep accurate and records (including adverse effects) and billing, iv) are accountable, v) obtain informed consent from participants, vi) terminate services when appropriate, and vii) provide adequate supervision and coaching throughout sessions. More detail regarding the scope and aims of the ISNR can be found at: http://www.isnr.net/about-isnr/code-of-ethics.cfm.

## Conclusion

Overall, modest positive improvements to a number of cognitive domains were identified following NFT initiation in a stroke population. Due to the satisfactory methodological quality and low level of evidence, results should be interpreted with caution. Future studies should strive to design high calibre NFT protocols including the use of randomized controlled trial study designs (with participant and administrator blinding) with large sample sizes to confirm current findings. Investigators may also wish to incorporate a waitlist control group within their study design thereby allowing interested participants access to NFT sessions following the treatment restriction period. Investigators should aim to include participants falling within acute, sub-acute and chronic phases of stroke recovery. Thorough descriptions of participants should be provided to examine the role of all potential confounding factors contributing to the success of NFT treatment. Additional studies should seek to replicate NFT protocol design (i.e. total # of sessions, frequency, intensity, feedback sites and NFT paradigms) to determine dose-response. Study designs should include plans for long-term follow-up with the use of objective and validated measures; this will help determine the longevity of NFT treatment effects. Lastly, potential participants are encouraged to consult BCIA accredited professionals when seeking and receiving NFT treatment.

## Supporting information

S1 File Search log. Table A. Primary Searches. Table B. Secondary Searches. Table C. Tertiary Searches. Table D. Journal Searches.
(PDF)

S2 File PRISMA checklist (PDF)

## References

[1] Statistics Canada. The 10 leading causes of death, 2011. URL: http://www.statcan.gc.ca/pub/82-625-x/2014001/article/11896-eng.htm

[2] Staines WR, McIlroy WE, Brooks D. Functional impairments following stroke: Implications for rehabilitation. Current Issues in Cardiac Rehabilitation and Prevention. URL: http://www.cacpr.ca/information_for_public/documents/Article3.pdf

[3] The Heart and Stroke Foundation. Stroke Report 2014: Together against a riding tide. June 2014. URL: heartandstroke.ca/strokereport2014

[4] National Stroke Association. Post-Stroke Conditions. URL: http://www.stroke.org/we-can-help/survivors/stroke-recovery/post-stroke-conditions

[5] Ottawa panel evidence-based clinical practice guidelines for post-stroke rehabilitation. Topics in Stroke Rehabilitation. 2006;13(2):1–296. doi: 10.1310/3TKX-7XEC-2DTG-XQKH 16939981 10.1310/3TKX-7XEC-2DTG-XQKH

[6] Doppelmayr M, Nosko H, Pecherstorfer T, Fink A. An attempt to increase cognitive performance after stroke with neurofeedback. Biofeedback. 2007;35(4)126–130.

[7] National Institute of Neurological Disorders and Stroke. Post-Stroke Rehabilitation Fact Sheet. April 2011. URL: http://www.ninds.nih.gov/disorders/stroke/poststrokerehab.htm

[8] Hasbini MJ, Underhill SM, de Erausquin G, Goldberg MP. Synapse loss and regeneration: a mechanism for function decline and recovery after cerebral ischemia? The Neuroscientist. 2000;6(2): 110–119.

[9] Bayona NA, Bitensky J, Teasell R. Plasticity and reorganization of the brain post stroke. Topics in Stroke Rehabilitation. 2005;12(3):11–26. doi: 10.1310/6AUM-ETYW-Q8XV-8XAC 16110424 10.1310/6AUM-ETYW-Q8XV-8XAC

[10] Bayona NA, Bitensky J, Teasell R. Plasticity and reorganization of the uninjured brain. Topics in Stroke Rehabilitation. 2005;12(3):1–10 doi: 10.1310/A422-G91U-Q4HB-86XC 16110423 10.1310/A422-G91U-Q4HB-86XC

[11] Liu Y, Yan X, Sun Z, Chen B, Han Q, et al Flk-1+ adipose-dervied mesenchymal stem cells differentiate into skeletal muscle satellite cells and amerliorate muscular dystrophy in mdx mice. Stem Cells Dev. 2007;16(5):695–705. doi: 10.1089/scd.2006.0118 17999592 10.1089/scd.2006.0118

[12] Rickhag M, Wieloch T, Gido G, Elmer E, Krogh M, et al Comprehensive regional and temporal gene expression profiling of the rat brain during the first 24 h after experimental stroke identifies dynamic ischemia-induced gene expression patterns, and reveals a biphasic activation of genes in surviving tissue. Journal of Neurochem. 2006;96:14–29. 10.1111/j.1471-4159.2005.03508.x 16300643

[13] Pedersen P, Jorgensen H, Nakayama H, Raaschou HO, Olsen TS. Aphasia in acute stroke: incidence, determinants, and recovery. Ann Neurol. 1995;38:659–666. doi: 10.1002/ana.410380416 7574464 10.1002/ana.410380416

[14] Heir D, Mondlock J, Caplan L. Recovery of behavioral abnormalities after right hemispheric stroke. Neurology. 1983; 33:345–350. 6681880 10.1212/wnl.33.3.345

[15] Nakayama H, Jorgensen H, Raaschou H, Olsen T. Recovery of upper extremity function in stroke patients: the Copenhagen Stroke Study. Arch Phys Med Rehabilitation. 1994;75:394–398. 10.1016/0003-9993(94)90161-9 8172497

[16] Duncan P, Lai S, Keighley J. Defining post stroke recovery: implications for design and interpretation of drug trials. Neuropharmocology. 2000;39:835–841. 10.1016/s0028-3908(00)00003-4 10699448

[17] Cicerone KD, Dalberg C, Kalmer K, Langenbahn DM, Malec JF, et al Evidence-based cognitive rehabilitation: Recommendations for clinical practice. Arch Phys Med Rehabil. 2000;81(12):1596–615. doi: 10.1053/apmr.2000.19240 11128897 10.1053/apmr.2000.19240

[18] Koehler R, Wilhelm EE, Shoulson I (Eds.). Cognitive Rehabilitation Therapy for Traumatic Brain Injury. Washington DC: The National Academies Press 2011. (pg29)

[19] Nelson LA. The role of biofeedback in stroke rehabilitation: past and future directions. Topics in Stroke Rehabilitation. 2007;14(4):59 doi: 10.1310/tsr1404-59 17698458 10.1310/tsr1404-59

[20] Kleim JA, Jones TA. Principles of experience-dependent neural plasticity: Implications for rehabilitation after brain damage. Journal of Speech, Language and Hearing Research. 2008;51:S225–S239. 10.1044/1092-4388(2008/018) 18230848

[21] Hammond D. What is neurofeedback? Journal of Neurotherapy. 2006;10(4):25–36.

[22] NeurOptimal: Advanced Brain Training Systems. 2017. Available from: https://neuroptimal.com/learn/dynamical-neurofeedback/.

[23] Duff J. The usefulness of quantitative EEG (QEEG) and neurotherapy in the assessment and treatment of post-concussion syndrome. Clinical EEG & Neuroscience. 2004;35(4):198–209. 15493535 10.1177/155005940403500410

[24] Othmer S. EEG biofeedback training for hyperactivity, Attention Deficit Disorder, specific learning disabilities, and other disorders. Association for Applied Psychophysiology and Biofeedback 24th annual meeting.

[25] Sterman MB, McDonald L. Effects of central cortical EEG feedback training on incidence of poorly controlled seizures. Epilepsia. 1978;19(3):207–222. 354919 10.1111/j.1528-1157.1978.tb04483.x

[26] Thompson L, Thompson M, Reid A. Neurofeedback outcomes in clients with Asperger’s Syndrome. Applied Psychophysiology and Biofeedback. 2010;35(1):63–81. doi: 10.1007/s10484-009-9120-3 19908142 10.1007/s10484-009-9120-3

[27] Coben R, Linden M, Myers TE. Neurofeedback for Autistic Spectrum Disorder: A review of the literature. Applied Psychophysiology and Biofeedback. 2010;35(1):83–105. doi: 10.1007/s10484-009-9117-y 19856096 10.1007/s10484-009-9117-y

[28] Breteler MHM, Arns M, Peters S, Giepmans I, Verhoeven L. Improvements in spelling after QEEG-based neurofeedback in Dyslexia: A randomized controlled treatment study. Applied Psychophysiology and Biofeedback. 2010;35(1):5–11. doi: 10.1007/s10484-009-9105-2 19711183 10.1007/s10484-009-9105-2 PMC2837193

[29] Kayiran S, Dursun E, Dursun N, Ermutlu N, Karanursel S. Neurofeedback intervention in Fibromyalgia Syndrome: A randomized, controlled rater blind clinical trial. Applied Psychophysiology and Biofeedback. 2010;35(4):293–302. doi: 10.1007/s10484-010-9135-9 20614235 10.1007/s10484-010-9135-9

[30] Putnam JA. EEG biofeedback on a female stroke patient with depression: A case study. Journal of Neurotherapy. 2001;5(3):27–28.

[31] Dehghani-Arani F, Rostami R, Nadali H. Neurofeedback training for opiate addition: improvement of mental health and craving. Applied Psychophysiology and Biofeedback. 2013;38(2):133–141. doi: 10.1007/s10484-013-9218-5 23605225 10.1007/s10484-013-9218-5 PMC3650238

[32] Moher D, Liberati A, Telzlaff J, Altman DG, The PRISMA Group (2009). *P*referred *R*eporting *I*tems for *S*ystematic *R*eviews and *M*eta-*A*nalyses: The PRISMA Statement. BMJ. 2009;339:b2535 doi: 10.1136/bmj.b2535 19622551 10.1136/bmj.b2535 PMC2714657

[33] Center Reviews and Dissemination. Systematic Reviews. University of York 2009.

[34] Khan KS, Kunz R, Kleijnen J, Antes G. Five steps to conducting a systematic review. Journal Of The Royal Society of Medicine. 2003;96:118–121. 12612111 10.1258/jrsm.96.3.118 PMC539417

[35] Simons M, Marks K. Guidelines for writing systematic reviews. Office of the Librarians at MacQuarie Univerity 2011.

[36] Glasziou P, Irwing L, Bain C, Colditz G. Systematic reviews in health care: A practical guide. University Press, UK, Cambridge 2001.

[37] Phillips B, Ball C, Sackett D, Badenoch D, Staus S, Haynes B. Oxford Centre for Evidence-based Medicine- Levels of Evidence (March 2009). URL: http://www.cebm.net/oxford-centre-evidence-based-medicine-levels-evidence-march-2009/

[38] Downs SH, Black N. The feasibility of creating a checklist for the assessment of the methodological quality both of randomised and non-randomised studies of health care interventions. J Epidemiol Community Health. 1998;52:377–384. 9764259 10.1136/jech.52.6.377 PMC1756728

[39] National Collaborating Centre for Methods and Tools (2008). Quality Checklist for Health Care Intervention Studies. Hamilton, ON: McMaster University 6 2010 URL: http://www.nccmt.ca/registry/view/eng/9.html.

[40] Deeks JJ, Dinnes J, D’Amico R, Sowden AJ, Sakarovitch C, Song F, et al Evaluating non-randomized intervention studies. Health Technol Assess. 2003;7(27):iii–x,1–173. 14499048 10.3310/hta7270

[41] Rozelle GR, Budzynski TH. Neurotherapy for stroke rehabilitation: a single case study. Biofeedback and Self-Regulation. 1995;20(3):211–28. 7495916 10.1007/BF01474514

[42] Bearden TS, Cassisi JE, Pineda M. Neurofeedback training for a patient with thalamic and cortical infarctions. Applied Psychophysiology & Biofeedback. 2003;28(3):241–53. 12964455 10.1023/a:1024689315563

[43] Cannon K, Sherlin L, Lyle R. Neurofeedback efficacy in the treatment of a 43-Year-old female stroke victim: a case study. Journal of Neurotherapy. 2010;14(2):107–21.

[44] Toppi J, Mattia D, Anzolin A, Risetti M, Petti M, Cincotti F, et al. Time varying effective connectivity for describing brain network changes induced by a memory rehabilitation treatment. Conference proceedings: Annual International Conference of the IEEE Engineering in Medicine and Biology Society IEEE Engineering in Medicine and Biology Society Annual Conference. 2014;2014:6786–9. 10.1109/EMBC.2014.6945186 25571554

[45] Mroczkowska D BJ, Rakowska A. Neurofeedback as supportive therapy after stroke. Case Report. Advances in Psychiatry and Neurology. 2014;23:190–201.

[46] Cho H-Y, Kim K, Lee B, Jung J. The effect of neurofeedback on a brain wave and visual perception in stroke: a randomized control trial. Journal of Physical Therapy Science. 2015;27(3):673–6. doi: 10.1589/jpts.27.673 25931705 10.1589/jpts.27.673 PMC4395689

[47] Jørgensen HS, Nakayama H, Raaschou HO, Vive-Larsen J, Støier M, Olsen TS. Outcome and time course of recovery in stroke. Part II: Time course of recovery. The copenhagen stroke study. Archives of Physical Medicine and Rehabilitation. 1995;76(5):406–12. 7741609 10.1016/s0003-9993(95)80568-0

[48] Unknown. Optimal stroke recovery with hyperbaric oxygen therapy. Stroke Recovery: Baromedical. Available from: http://baromedical.ca/medical-stroke-recovery.php.

[49] Unknown. Glossary; Stroke Engine: Canadian Partnership for Stroke Recovery. Available from: http://www.strokengine.ca/glossary/chronic-stage-of-recovery/.

[50] Kiran S. What is the nature of poststroke langauge recovery and reorganization? ISRN Neurology. 2012: Article ID 786872. 10.5402/2012/786872 PMC3540797 23320190

[51] Ammann BC, Knols RH, Baschung P, de Bie RA, de Bruin ED. Application of principles of exercise training in sub-acute and chronic stroke suvivors: a systematic review. BMC Neurology. 2014;14(167). 10.1186/s12883-014-0167-2 PMC4236657 25162455

[52] MacLehose RR, Reeves BC, Harvey IM, Sheldon TA, Russell IT, Black AM. A systematic review of comparisons of effect sizes derived from randomised and non-randomised studies. Health Technology Assessment. 2000;4(34):1 11134917

[53] Kirk L, Hammond DC. First, Do No Harm: Adverse Effects and the Need for Practice Standards in Neurofeedback. Journal of Neurotherapy. 2008;12(1):79–88.

[54] Matthews T. Neurofeedback Overtraining and the Vulnerable Patient. Journal of Neurotherapy. 2008;11(3):63–6.

[55] Hammond DC, Bodenhamer-Davis G, Gluck G, Stokes D, Harper SH, et al Standards of practice for neurofeedback and neurotherapy: a position paper of the International Society for Neurofeedback and Research. Journal of Neurotherapy. 2011;15:54–64.

[56] Research ISfN. Code of Ethical Principles & Professional Conduct 2012. Available from: http://www.isnr.net/about-isnr/code-of-ethics.cfm.

