# Supplementary material for: Neurofeedback as a form of cognitive rehabilitation therapy following stroke: A systematic review
Source: PLoS One. 2017 May 16;12(5):e0177290. doi: 10.1371/journal.pone.0177290 (PMC5433697; doi:10.1371/journal.pone.0177290)
Supplement: S1 File — Table A. Primary Searches. Table B. Secondary Searches. Table C. Tertiary Searches. Table D. Journal Searches. (PDF) [file pone.0177290.s001.pdf]

S1 File. Search Log

Table A. Primary Searches

Date	Search Terms	Database	Results	Results (without duplicates)	# of Full Articles Identified for Further Screening	Exclusion reasons	# Citations included in review
July 17/2015	1. exp Brain/ or exp Cognition Disorders/ or exp Brain Damage, Chronic/ or cognitive rehabilitation therapy.mp. or exp Cognitive Therapy/ or exp Stroke/<br>2. exp Stroke/rh, th [Rehabilitation, Therapy]<br>3. neurofeedback.mp. or exp Biofeedback, Psychology/ or exp Neurofeedback/ or exp Electroencephalography/<br>4. 1 and 2 and 3	OVID Embase & Embase Classic	381			1.Not a stroke population (n=5)<br>2.Motor/balance/ postural/dysphagia intervention (n=8)<br>3.No intervention/ for monitoring purposes only (n=3)<br>4.Did not include NFT with a cognitive impairment target (n=4)<br>5.Other (daytime sleepiness intervention) (n=1)<br>6.Editorial review (n=1)<br>7.Not available in English (n=5) (German n=1, Chinese n = 2, Spanish n=1, Portuguese n=1)<br>Total Articles Excluded: 27	
		OVID Medline	296	**632**	**31**		**4**
		OVID Health Star	315				
	(((stroke OR cerebral vascular accident OR brain infarct OR brain haemorrhage)) AND (cognitive rehabilitation therapy OR cognit* OR CRT OR neurotherapy)) AND (neurofeedback OR NFT OR biofeedback OR brain-computer interface OR BCI)	PubMed	83				

S1 File. Search Log

Table B. Secondary Searches

Date	Search Terms	Database	Results	Results (without duplicates)	# of Full Articles Identified for Further Screening	Exclusion Reasons	# Citations Included In Review
Aug 1/2015	1. exp Stroke/ or stroke.mp.<br>2. neurofeedback.mp. or exp Biofeedback, Psychology/ or exp Neurofeedback/ or exp Electroencephalography/<br>3. cognitive therapy.mp. or exp Cognitive Therapy/<br>4. attention.mp. or exp Attention/<br>5. exp Memory/ or memory.mp. or exp Memory Disorders/<br>6. exp Stroke/ or neglect.mp.<br>7. executive function.mp. or exp Executive Function/ or exp Brain Damage, Chronic/<br>8. exp Communication Disorders/ or exp Communication/ or communication.mp.<br>9. aphasia.mp. or exp Aphasia/<br>10. exp Brain/ or motor impairment.mp. or exp Stroke/<br>11. balance.mp. or exp Postural Balance/<br>12. upper limb.mp. or exp Upper Extremity/<br>13. exp Movement/ or movement.mp.<br><br>14. (1 and 2 and 4) not 10 not 11 not 12 not 13<br>15. (1 and 2 and 5) not 10 not 11 not 12 not 13<br>16. (1 and 2 and 6) not 10 not 11 not 12 not 13<br>17. (1 and 2 and 7) not 10 not 11 not 12 not 13<br>18. (1 and 2 and 8) not 10 not 11 not 12 not 13<br>19. (1 and 2 and 9) not 10 not 11 not 12 not 13	OVID Medline<br><br><br><br>OVID HealthStar<br><br>*recorded and exported results utilized hits from line 14 – 19*	111<br><br><br><br>102	**431**	**89**	1.Not a stroke population (n=7)<br>2.Motor/balance/postural related/dysphagia (n=9)<br>3.No intervention// for monitoring purposes only (n=21)<br>4.Did not include NFT intervention with a cognitive impairment target (n=6)<br>5.Not available in English (n=7) (Spanish n=1, Portuguese n=1, German n=1, Croatian n=1, Russian n=2, Polish n=1)<br>6.Editorial review/ systematic (n=10)<br>7. Intervention included stimulation (n=12)<br>8.Intervention included pharmacological method (n=3)<br>9.Could not retrieve article (n= 5)<br>10.Multi-reason exclusion (n=9)<br>Total Articles Excluded: 89	**0**
	20. 1 and 2 and 3 and 4<br>21. 1 and 2 and 3 and 5<br>22. 1 and 2 and 3 and 6<br>23. 1 and 2 and 3 and 7<br>24. 1 and 2 and 3 and 8<br>25. 1 and 2 and 3 and 9<br>*corresponding search terms are indicated by the above search as indicated by search #*	OVID Embase & Embase Classic<br><br>*above search method narrowed search too much, therefore it was modified as indicated*	175				
	((((((cogniti* OR attention OR memory OR perception OR neglect OR planning OR executive function OR communication OR language OR speech)) AND (stroke OR cerebral vascular accident OR brain infarct OR brain hemorrhage OR brain bleed)) AND (neuro feedback OR NFT OR brain-computer interface OR evoked response potential OR ebrp OR qeeg OR EEG OR MEG OR brain biofeedback OR brain waves)) NOT (motor impairment OR movement OR balance OR extremity OR limb))) AND (cognitive rehabilitation OR therapy OR CRT)	PubMed	337				

S1 File. Search Log

Table C. Tertiary Searches

Date	Search Terms	Database	Results	Citations Included
Jun 27/2015	Advanced search function: With all words: stroke, neurofeedback, cognitive Without the words: motor, movement, imagery	Google Scholar *excluded patents*	2	**0**
	Advanced search function: With all words: stroke, neurofeedback, With at least one word: cognitive, cognition, speech, memory, executive, function, communication, attention, perception, Without words: motor, movement, arm, limb, paralysis	*no date filter* *filter to: words in title of article *	5	**1**
Jun 27 /2015	"neurofeedback following stroke"	U of T Online Library Catalogue	391	**2**
Jun 27/2015	neurofeedback AND stroke	The Cochrane Database of Systematic Reviews (2012 – present)	10	0
		Clinicaltrials.gov (grey literature)	3	0

Note: Google Scholar searches were modified using Without Words function in advanced search settings. Additional filter was used " words in title of article " .

S1 File. Search Log

Table D. Journal Searches

Date	Journal Name	Dates Searched	# of Additional Citations Included
March 11/2015	Biofeedback	Spring 2005-Spring 2014	0
March 12/2015	Applied Psychophysiology and Biofeedback	2005-December 2014	0
	Brain and Cognition	2005-March 2015	0
	Journal of Clinical Neurophysiology	2005-February 2015	0
	Clinical EEG and Neuroscience (formerly Clinical Electroencephalography)	2005-Nov/Dec 2014	0
	Clinical Neurophysiology (formerly Electroencephalography and Clinical Neurophysiology)	2005-April 2015	0
	Topics in Stroke Rehabilitation	2005-February 2015	0
	Stroke	2005-March 2015	0
	Stroke Research and Treatment	*2010-2015	0
	International Journal of Stroke	*2006-February 2015	0
April 1/ 2015	Journal of Neurotherapy	2005-2013	0
July 28/2015	Biofeedback	Spring 2014-Spring 2015	0
	Applied Psychophysiology and Biofeedback	December 2014-June 2015	0
	Brain and Cognition	March 2015-July 2015	0
	Journal of Clinical Neurophysiology	February 2015-July 2015	0
	Clinical EEG and Neuroscience	December 2014-July 2015	0
	Clinical Neurophysiology	April 2015-July 2015	0
	Topics in Stroke Rehabilitation	February 2015-June 2015	0
	Stroke	March 2015-August 2015	0
	Stroke Research and Treatment	2015	0
	International Journal of Stroke	February 2015-August 2015	0
	Journal of Neurotherapy	January 2013-October 2013	0

Note: All journals listed here published issues past 2005. Hand searches started at the first issue published in that year.
* journals didn't have issues prior to date indicated and were reviewed from inception issue.
